# Potential Role of L-Carnitine in Autism Spectrum Disorder

**DOI:** 10.3390/jcm10061202

**Published:** 2021-03-13

**Authors:** Alina Kępka, Agnieszka Ochocińska, Sylwia Chojnowska, Małgorzata Borzym-Kluczyk, Ewa Skorupa, Małgorzata Knaś, Napoleon Waszkiewicz

**Affiliations:** 1 Department of Biochemistry, Radioimmunology and Experimental Medicine, The Children’s Memorial Health Institute, 04-730 Warsaw, Poland; a.kepka@ipczd.pl (A.K.); e.skorupa@ipczd.pl (E.S.); 2 Faculty of Health Sciences, Lomza State University of Applied Sciences, 18-400 Lomza, Poland; sylwiacho3@gmail.com (S.C.); knass@wp.pl (M.K.); 3 Department of Pharmaceutical Biochemistry, Medical University of Bialystok, 15-089 Bialystok, Poland; malgorzata.borzym-kluczyk@umb.edu.pl; 4 Department of Psychiatry, Medical University of Bialystok, 15-089 Bialystok, Poland; napwas@wp.pl

**Keywords:** autism spectrum disorder (ASD), biomarkers, L-carnitine, mitochondria

## Abstract

L-carnitine plays an important role in the functioning of the central nervous system, and especially in the mitochondrial metabolism of fatty acids. Altered carnitine metabolism, abnormal fatty acid metabolism in patients with autism spectrum disorder (ASD) has been documented. ASD is a complex heterogeneous neurodevelopmental condition that is usually diagnosed in early childhood. Patients with ASD require careful classification as this heterogeneous clinical category may include patients with an intellectual disability or high functioning, epilepsy, language impairments, or associated Mendelian genetic conditions. L-carnitine participates in the long-chain oxidation of fatty acids in the brain, stimulates acetylcholine synthesis (donor of the acyl groups), stimulates expression of growth-associated protein-43, prevents cell apoptosis and neuron damage and stimulates neurotransmission. Determination of L-carnitine in serum/plasma and analysis of acylcarnitines in a dried blood spot may be useful in ASD diagnosis and treatment. Changes in the acylcarnitine profiles may indicate potential mitochondrial dysfunctions and abnormal fatty acid metabolism in ASD children. L-carnitine deficiency or deregulation of L-carnitine metabolism in ASD is accompanied by disturbances of other metabolic pathways, e.g., Krebs cycle, the activity of respiratory chain complexes, indicative of mitochondrial dysfunction. Supplementation of L-carnitine may be beneficial to alleviate behavioral and cognitive symptoms in ASD patients.

## 1. Introduction

Autism spectrum disorder (ASD) involves different neurodevelopment disorders with heterogeneous etiology. ASD clinical manifestations start from the third year of life. According to the International Classification of Diseases, Tenth Revision (ICD-10) and The Diagnostic and Statistical Manual of Mental Disorders, Fifth Edition (DSM-5), ASD is defined by a disturbance in communications/social interactions and stereotypically repeated behavior. ASD diagnosis is supplemented by evaluation of verbal communication and intellect level [1]. Interactions between genetic and environmental factors affecting mother before and during pregnancy, e.g., chemicals (arsenate, mercury, lead, polychlorinated biphenyls, toluene, and pesticides), perinatal injuries, infections during pregnancy, hypoxia, preterm delivery, and intensive antibiotic therapies during pregnancy disturbing embryogenesis, are thought to significantly impact ASD occurrence [2]. Structural changes in the brain (depending on sex) have been shown to be significantly related to autistic features [3].

ASD is genetically determined, with many familial patterns of inheritance and an estimated up to 1000 potentially related genes, pointing to a complex genetic etiology. The majority of defective genes connected with ASD are engaged in synaptogenesis, transformations of neurotransmitters, neurometabolism, and mitochondria dysfunction [3]. Some 10–15% of ASD patients have other monogenic disorders: fragile X chromosome syndrome, nodular induration, Down’s syndrome, phenylketonuria, neurofibromatosis type I, and Angelman syndrome [4]. More than 70% of patients with ASD have other diseases: epilepsy (30%), gastrointestinal problems (9–70%), immunological regulatory problems (38%), and sleep disorders (50–80%) [5]. Symptoms of mitochondria dysfunction such as mutations in mitochondrial DNA and deletion and mutations in mitochondrial and nuclear genes are also found in ASD [6].

**Figure 1:**
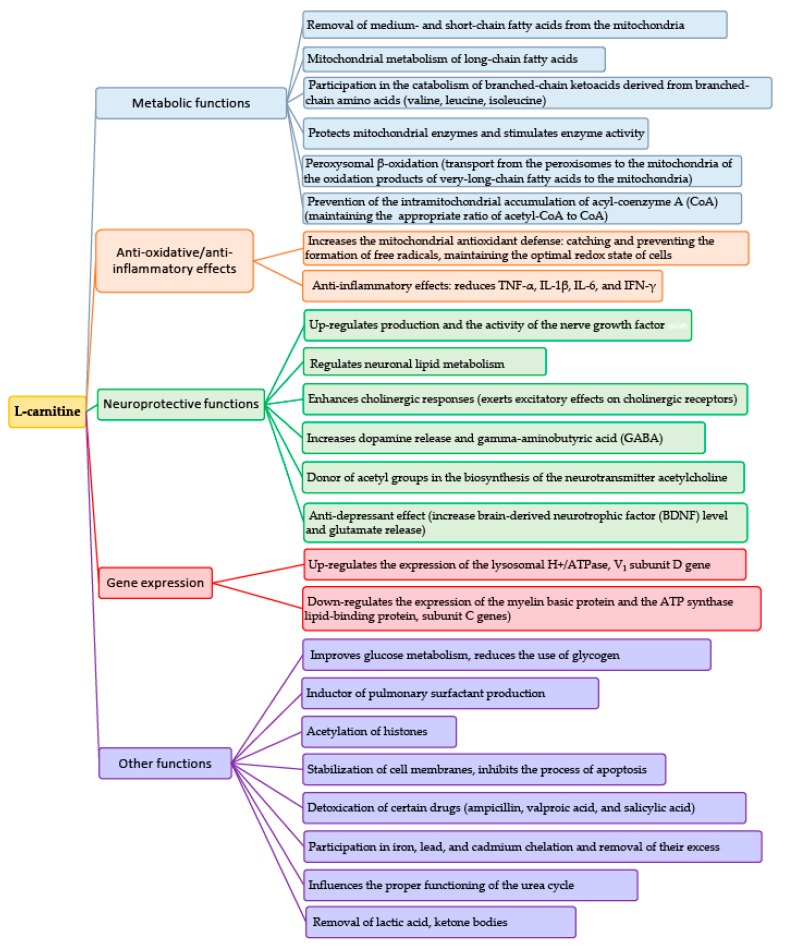
Roles of L-carnitine.

The occurrence of abnormal biomarkers indicating mitochondrial dysfunction is more common in patients with ASD than in the general population. L-carnitine is necessary to transfer long-chain fatty acids from the cytoplasm to mitochondria through the internal mitochondrial membrane for β-oxidation [7]. L-carnitine (LC) is necessary for the correct functioning of fetal nervous stem cells (NSCs); their duplications and differentiation is one of three cellular lines covering: neurons, oligodendrocytes, and astrocytes. Disordered β-oxidation of long-chain fatty acids in the NSCs of the developing brain caused by L-carnitine deficiency increases the risk of developing ASD in the fetus [8]. LC is involved in the catabolism of endogenous organic acids, including ketoacids derived from branched-chain amino acids (valine, leucine, and isoleucine) and renal excretion of endogenous and exogenous acids derived from xenobiotics, such as some antibiotics and valproic and salicylic acids [9]. Complexes of branched-chain ketoacids connected with carnitine are transported to the liver, where they are oxidized or used as substrates for glyconeogenesis. Carnitine chelates cadmium, lead, and ferric cations, inhibiting free radical production. Anemia due to iron deficiency associated with hyperlipidemia may be related to iron chelation by carnitine [9]. LC protects the thiol groups of proteins against oxidative action of H_2_O_2_ and free radicals (i.e., any molecular species capable of independent existence that contain an unpaired electron in an atomic orbital) and decreases peroxidative damage of the unsaturated fatty acids (FAs) built into membrane phospholipids that stabilize cell membranes and ion canals [9]. In neonates, carnitine seems to act as an inductor of pulmonary surfactant production via phospholipids repair activity [7,10]. Deficiency and deregulation of carnitine metabolism accompanied by slight increases in lactate and serum ammonia in ASD are symptoms of mitochondrial dysfunction [11] (Figure 1).

L-carnitine deficiency in ASD may be caused by a deletion in the *TMLHE* gene, coding ε-N-trimethyllysine dioxygenase (TMLD), which is involved in L-carnitine synthesis, resulting in an increased risk of ASD symptoms in children [12]. ASD children show low concentrations of free L-carnitine, as L-carnitine is connected with long-chain fatty acids in tissues and serum. Increases in the concentrations of long- and very-long-chain fatty acids, suggesting their impaired β-oxidation, were reported in the serum of ASD children [13,14]. The efficiency of mitochondria oxidation reflects direct (lactate, pyruvate, and lactate/pyruvate ratio) and indirect (creatine kinase (CK) activity, L-carnitine concentration, activities of alanine (ALT) and aspartate (AST) aminotransferases and ammonia concentration) markers, which may be useful in ASD diagnostics [15].

## 2. Mitochondrial Dysfunction in ASD

Mitochondria are organelles present in every cell (with the exception of erythrocytes), named the cellular power plant, playing a key role in the energy metabolism of the cell. Mitochondria host machinery uses mostly carbohydrates and fats for the production of energy as ATP. ATP provides 90% of the energy necessary for cells and tissues [16]. The majority of ATP is generated in three carboxylic acid cycles and β-oxidation in the mitochondrial transport chain of electrons, consisting of I–IV complexes, ubiquinone (coenzyme Q10), and cytochrome C [17]. The energy produced in the mitochondria is responsible for cellular respiration, cell division and apoptosis, maintaining homeostasis (by calcium storage), enzymatic regulation, and maintaining the proper redox state of the cells [18]. Mitochondria are engaged in the proliferation and maturation of the nervous stem cells and synaptic functions, playing a key role in the development and regulation of synaptic activity [19]. Mitochondria are the main producer of reactive oxygen species (ROS) and free oxygen radicals, which include ozone (O_3_), singlet oxygen (^1^O_2_), superoxide radical anion (O_2_^•−^), hydrogen peroxide (H_2_O_2_), and hydroxyl radical (HO^•^), which are extremely chemically reactive and can react with virtually all substances in the cell [20]. Neuroimaging, in vitro, and post-mortem brain studies showed an increased prevalence of mitochondrial dysfunction in ASD of differing severities. Mitochondrial disturbances in ASD may be caused by mitochondrial, chromosomal, and nuclear DNA disorders [21]. Large amounts of harmful mutations of mitochondrial DNA in ASD children were reported compared to their family members without ASD disturbance [22].

Mitochondrial dysfunction concerns organs requiring large amounts of energy, such as the heart, skeletal muscles, intestines, hormonal system, and brain. Many studies have documented the prevalence of various co-occurring psychiatric and behavior dysregulation symptoms in children and adults with ASD. ASD symptoms are connected with pathological redox reactions and abnormal mitochondrial metabolism of differing severities. Many ASD children present new types of mitochondria dysfunction, e.g., metabolic disturbances (inborn errors of amino acids metabolism in maple syrup disease (MSUD), dysregulation of the immunological system (ASD development may be connected with an increase in proinflammatory cytokines in natal-and perinatal periods) and/or inflammation in the nervous system, and oxidative stress (increased production of the reactive oxygen species). Mitochondrial damage may be caused by exposure to toxic substances such as heavy metals (mercury, lead), toxins, and air pollution. Certain drugs, such as thalidomide, valproic acid, retinoic acid, pathogenic bacteria, and viruses, can lead to mitochondrial dysfunction [23,24]. Current epidemiological data indicate an increasing trend in the prevalence of ASD. The estimated median prevalence for North America and Europe is 65.5/10,000 and 61.9/10,000, respectively [25]. Current estimates for Poland are based on the register of the National Health Fund, and the median frequency of ASD is 17.6/10,000 in children aged 0–18 years [26]. Early diagnosis of ASD anomalies is important because it may increase the number of screening tests, enable correct treatment, and introduce strategies for the prevention of autistic diseases. For example, identification of toxins that may be associated with ASD may help with ASD prevention [27]. Understanding metabolic dysfunctions in ASD and identification of the specific markers, e.g., glutathione, vitamin E, tetrahydrobiopterin, and antioxidative enzymes such as glutathione peroxidase, paraoxonase, and superoxide dismutase, may direct ASD treatment toward specific metabolic disturbances [23,28]. It was reported that the developing child brain uses a particularly large amount of energy, mostly in the form of glucose, which is used for ATP production in the brain mitochondria in the Krebs cycle and oxidative phosphorylation. Therefore, disturbance of any aspect of the mitochondrial energy program may disturb the development of ASD in children [29,30]. For example, a deficit in complex IV of the mitochondrial respiratory chain (in comparison to the control group) was more often found in children with ASD and coexisting epilepsy [31]. It was reported that 80% of ASD children might present disturbances in mitochondrial electron transport systems; this research was performed on immunological cells collected from autistic children [32]. Therefore, in ASD children, the combination of anti-epilepsy treatment with mitochondrial therapy may be beneficial [33,34]. Neurological and muscular disturbances (motor disturbances, convulsions, and excessive fatigue (weakness)) and laboratory tests (increased or decreased concentration of different biochemical markers in tissues, blood, and urine) can indicate mitochondrial damage [26,35]. Determination of reduced glutathione (GSH) is an example of a useful laboratory test in ASD. The GSH concentration in serum, as well as the brain of patients with ASD, is lower, but the ratio of oxidized glutathione (GSSG) to reduced glutathione is higher because of impaired GSH synthesis. Lowered ATP levels in the blood and brain are observed in ASD patients during autopsy. People with ASD show a significant lowering of oxidized nicotinamide adenine dinucleotides (NAD^+^) concentration in serum, with impaired NAD^+^/NADH ratio, which may indicate a disturbed energetic metabolism in their brains. Impaired or incorrect activities of glutathione peroxidase (GPx), catalase, superoxide dismutase (SOD), and antioxidative compounds in the blood and decreases in the antioxidative capacity of the above-listed markers are frequently detected in ASD [36,37].

## 3. Direct and Indirect Biochemical Markers of Mitochondrial Dysfunction

There are direct markers of mitochondrial dysfunction: lactate, pyruvate, lactate/pyruvate ratio, ubiquinone, alanine, alanine/lysine ratio, and acylcarnitine profiles, and indirect markers of mitochondrial function, such as creatine kinase (CK), free L-carnitine and total L-carnitine (TC), alanine aminotransferase (ALT), aspartate aminotransferase (AST), and ammonia [15,38]. Increased serum and brain concentrations of lactate, pyruvate, lactate/pyruvate ratio, alanine, and creatine kinase, at 31%, 14%, 28%, 8%, 47%, 35%, 46%, and 7%, respectively, and low carnitine were described in 90% of children with ASD in the review of Rossignol and Frye [38]. In another paper, Frye and Rossignol [39] described similar changes in 17.1–76.6% of ASD children. The changes involved increases in the serum concentration of lactate and lactate/pyruvate ratio by 28% and an increase in alanine by 36% of ASD children. Frye and Rossignol reported changed urinary tricarbonic acids excretion and changed profiles of acylcarnitines in the described group of ASD children, which suggested a significant increase in frequency (about 30–50%) of abnormal mitochondrial function in ASD persons than in classical mitochondrial disease, defined as severe impairment of mitochondrial respiratory chain complexes. Similar to Frye and Rossignol, Poling et al. described increases in serum concentration of lactate, pyruvate, and alanine; increased activity of AST, ALT, and CK; and hyperammonemia in ASD persons [40]. Although serum lactate is treated as a direct ASD marker, hyperlactacidemia may be a result of physical exercise or collection of blood (fist clenching and unclenching) [41]. Other potential markers of ASD include lactate dehydrogenase (LDH), caspase-7 (CASP7), GSH, and glutathione S-transferase (GST). According to Khemakhem et al. [42], CASP7 is the best biomarker of ASD, constituting 100% sensitivity and specificity between ASD patients and the control group, suggesting the use of CASP7 as the diagnostic marker for early diagnosis of ASD.

Damage to mitochondrial β-oxidation leads to increased microsomal ω-fatty acid oxidation, leading to urinary excretion of dicarboxylic acids: malate, fumarate, ethylmalonic acid, 3-methyl-glutaconic acid, and citric acid cycle intermediates. In a retrospective study of children with mitochondrial autism, 96% had markers of mitochondrial dysfunction in their blood: increased lactate level in 76% of the study group, an increase in pyruvate in 53%, an increased level of alanine in 36%, and CK activity was increased in 32% and 42% of the subjects who had abnormal organic acids in their urine [43]. Another study reported elevated plasma lactate levels, along with an increased lactate/pyruvate ratio in 20.3% of 69 patients with ASD; mitochondrial disease was diagnosed in five of these patients (7.2%), which appears to be a high-frequency in autism [44]. Participation of the mitochondrial aspartate/glutamate gene *SLC25A12* was not confirmed in mitochondrial dysfunction connected with ASD. Correia et al. [45] concluded that variability in *SLC25A12* was not responsible for the frequent occurrence of biochemical markers of mitochondrial dysfunction (lactate and pyruvate) in their ASD research group. This discrepancy in research may reflect the genetic heterogeneity in autism [45]. Similar results were reported by Weissman et al. [46], who described disorders of the respiratory chain and mitochondrial (mtDNA) mutations. Two patients had mtDNA mutations of similar pathogenicity, and four had mtDNA sequence variants of unclear pathogenicity out of 25 tested children [46].

### L-Carnitine as a Specific Marker of Mitochondrial Function

Metabolic processes occurring in mitochondria, e.g., β-oxidation of fatty acids, may be disturbed. Free carnitine and acylcarnitine profiles in dried blood spots may be biomarkers of the pathological metabolism of fatty acids in patients with ASD. The profile from the non-invasively collected dried blood spot may support the diagnosis of metabolic ASD children aged ≤ five years and may be a potential diagnostic method complementing and improving the diagnosis in the early-stage of ASD development [47]. Significant increases in the concentrations of short- and long-chain acylcarnitines, but not in average-chain fatty acids carnitines, are shown in ASD persons. A similar panel of incorrect acylcarnitines was found in nervous rat tissue after induction of ASD [14]. Among 45 analyzed metabolites, nine (20%) were significantly increased in patients with ASD, including short-chain acylcarnitines: C2 and C4DC/C5OH long-chain acylcarnitines: C10, C12, C14:2, C16, C16:1, and C18:1; and citrulline.

Recommendations for the evaluation of the accuracy and sensitivity of analyzed biomarkers in ASD diagnostics were presented by Frye et al. [48]. A grade of recommendation (GOR) was recommended to allow estimation of biomarker quality using the Oxford Centre for Evidence-Based Medicine scale. For each clinical study that described a biomarker, a level of evidence (LOE) ranging from one to five was assigned. From these studies, a GOR ranging from A (solid evidence) to D (limited, inconsistent, or inconclusive evidence) was derived [48]. Frye et al. claimed that metabolic biomarkers, including methylation-redox, have 97% precision (sensitivity 72% and specificity 63%); acylcarnitine and amino acids precision have 69% precision (sensitivity 73% and specificity 63%). Occurrence and GOR (B,C) for particular substances are as follows: amino acids 17% (C); methylation-redox 98–97% (B); electron transport chain (ETC.) by buccal swab 62–64% (B); lactate 27–15% (B); pyruvate 7–20% (B); lactate/pyruvate ratio 28% (C); alanine 2–8% (B); alanine/lysine ratio 16% (C); acyl-carnitine elevations 17% (C). L-carnitine and its acetylated derivatives (free carnitine, isovalerylcarnitine (C5), and octanoyl carnitine (C8)) were positively associated with ASD, and two (methylmalonyl carnitine (C4DC) and adipylcarnitine (C6DC)) were inversely associated with later diagnosis of ASD; GOR C was assigned to them [48].

According to Rossignol and Frye, indirect biochemical markers of mitochondrial dysfunction appear to be abnormal in the general ASD population, occurring at a much higher frequency than direct biomarkers [38]. Rossignol and Frye recommended a screening algorithm of mitochondria function to evaluate the following parameters of blood collected before breakfast: lactate, pyruvate, coenzyme Q10, CK, AST, ALT, AST/ALT ratio, glucose, ammonia, concentration of amino acids, CO_2_ (preferably in arterial blood), L-carnitine (free and total), and acylcarnitines profile in a dried blood spot [38].

## 4. Physiological Properties of L-Carnitine and Acetyl-L-Carnitine

L-carnitine (2-hydroxy-4-trimethylammonium butyrate) is a small (162 Da) polar compound that exists as a double ion in physiological pH. Intracellular and extracellular carnitine may be present as either non-esterified free carnitine (FC) or as esters of short-, medium-, or long-chain organic and fatty acids. LC plays many important roles in the intracellular functions of the body, with its most important role as a contributor to cellular energy metabolism. The primary functions of FC and acylcarnitines (AC), mainly acetyl-L-carnitine (ALC) in humans, are the transport of long-chain fatty acids (FAs) to the mitochondrial matrix and maintaining the mitochondrial homeostasis of coenzyme A (CoA) during mitochondrial oxidation of these acids [49]. CoA is necessary for activation and oxidation of the FAs from adipose tissue for ATP synthesis in the mitochondria. FAs oxidation reduces glucose oxidation in the tissues where glucose is not an essential fuel. Fatty acids are a very efficient source of human energy. The amount of energy from the total oxidation of FAs is 37.7 kJ/g, compared to 16.7 kJ/g from protein or carbohydrates. Carnitine also participates in the detoxication processes of toxic exogenous compounds (e.g., some xenobiotics, including ampicillin, valproic acid, and salicylic acid), which are excreted by the kidneys when they are combined with carnitine [9]. The next most important role of carnitine is contributing to the catabolism of branched-chain ketoacids derived from branched-chain amino acids (valine, leucine, and isoleucine). L-carnitine also inhibits free radicals production and demonstrates antioxidant action [9].

LC has antioxidant effects and optimizes the functions of the complex of mitochondrial enzymes [50]. Carnitine also significantly increases dopamine levels in the cortex, hippocampus, and striatum of the rat brain [51].

### 4.1. L-Carnitine Content in Food

Red meat is the richest source of L-carnitine in adults and milk in infants and children [7], whereas plants contain only traces of carnitine [52] (Table 1).

The standard healthy human diet meets about three-quarters of the requirement for L-carnitine; the remaining one-quarter is synthesized in the human body from lysine (creating carnitine carbon skeleton) and methionine (origin of N-methyl groups) with the participation of ascorbic acid, niacin, pyridoxine, and Fe^+2^ [53] in the liver, kidneys, brain [10], and placenta [54]. Nutritional carnitine is actively (sodium-dependent) and passively transported from the intestinal content into enterocytes, with 54–86% bioavailability, depending on the amount of carnitine in the meal. In vegetarians adapted to diets with low carnitine, concentration and bioavailability are higher, around 66–86%; in individuals who prefer red meat in carnitine-rich diets, the bioavailability is around 54–72% [55]. The bioavailability of carnitine from diet supplements is much lower than from the general diet, reaching only 14–18%. The organism maintains carnitine homeostasis; with the increase in carnitine consumption, there is a decrease in carnitine absorption [56]. Absorbed carnitine appears mainly in the portal circulation; it is passively secreted into the bile, mainly in the form of acylcarnitine esters (greater than 66% of total carnitine) and long-chain acylcarnitine esters, amounting to 30–50% of the total bile carnitine [56]. Kidneys play the main role in carnitine and its ester homeostasis [22]. Carnitine is not metabolized in the human organism, but it undergoes filtration in renal tubules and is almost totally (98–99%) reabsorbed in the renal tubules. Transporter OCTN2, named CT1 (carnitine transporter 1), plays a key role in renal carnitine reabsorption in symport with two sodium cations. Renal OCTN2 activity is inhibited by short- and long-chain acylcarnitines [57].

### 4.2. Health Risks from High Amounts of L-Carnitine

Consumed, but not absorbed, carnitine is degraded, mainly in intestinal microorganisms to trimethylamine (TMA), which is a non-toxic substance with a very unpleasant fish smell; then, it reaches the liver where it is transformed to toxic trimethylamine N-oxide (TMNO, TMAO), which is excreted with urine, and γ-buterobetaine is eliminated mainly in feces. In humans, TMNO in urine accounts for 8–39% and γ-buterobetaine in feces to 0.1–8% of the total dietary carnitine [58]. TMA is produced in the intestines not only from L-carnitine but also from betaine, buterobetaine (GBB), choline, and other compounds containing choline, which are present in the typical diet (red meat, eggs, poultry, fish, and dairy products). Previously, TMA and TMNO were considered non-toxic substances, but recently they have been considered potentially carcinogenic agents because of possible transformation to N-nitrosodimethylamine (NDMA). Current research has proven that TMA and TMNO are compounds that favor the occurrence of cardiovascular disease and atherosclerosis and inhibit reverse cholesterol transport [59]. However, clinical implications of TMNO in the central nervous system have not yet been documented, but TMNO is present at detectable levels in the cerebrospinal fluid (CSF). In the small tested groups of subjects, TMNO levels in CSF were apparently unrelated to the diagnosed neurological disorders such as Alzheimer’s disease (AD) [60]. Recently, increased concentration of TMNO in the plasma of ASD patients was reported, which correlated with an intensification of ASD symptoms. Plasma levels of TMNO, choline, and betaine were higher in ASD patients than in healthy people. Therefore, the authors suggested that TMNO is a useful biomarker of ASD [61]. Elevated L-carnitine levels increase the risk of disease if TMNO levels are also elevated. A diet rich in animal products (containing carnitine and choline) changes the intestinal microflora by increasing the synthesis of TMAO. Additionally, another clinical study reported that disturbed TMNO metabolism and increased TMNO concentration in the serum/plasma were connected with renal diseases, cardiovascular diseases, diabetes type 2, and neurological disturbances [62].

### 4.3. Acetyl-L-Carnitine and the Carnitine Transporter OCTN2

L-carnitine is acetylated in the human intestine to active acetyl-L-carnitine (γ-trimethyl-β-acetylbutyre-betaine (ALC)). Since ALC is more easily transferred through intestinal serous membranes than non-acetylated L-carnitine, the intracellular acetylation of carnitine may facilitate its diffusion across the serous membrane. LC and its short-chain fatty esters do not connect with plasma proteins; though blood cells contain LC, the speed of LC distribution between erythrocytes and plasma is very slow [63]. In the circulation, about 75% of LC occurs in the free state, 15% as ALC, and the remaining 10% as esters of carnitine with other acids (e.g., propionyl-L-carnitine) [64]. In human tissues, L-carnitine is localized mainly in skeletal and cardiac muscles (98%), with only about 1.5% in the liver, kidneys, and brain, and in plasma, about 0.5–1% [7]. In the adult brain, about 80% of carnitine exists as FC, 10–15% as ALC and less than 10% as long-chain acylcarnitines [65]. LC is absorbed to the cells mainly with the help of organic cation/carnitine transporter 2 (OCTN2), occurring in skeletal muscles, heart, fibroblasts, placenta, renal tubules, and brain (located mainly in the cells of the endothelium of capillaries creating the blood–brain barrier, in astrocytes, and in neurons). OCTN2 is a unique transporter with a dual-mode of transport as both Na^+^-independent organic cation transporters and Na^+^-dependent and high-affinity carnitine transporters [57]. Defects in the carnitine transporter (OCTN2), which is coded by the SLC22A5 gene, create primary carnitine deficiency, expressed as low urinary carnitine excretion and low blood and tissues carnitine level, which may be a risk factor of ASD. The next protein transporting carnitine to the brain and to its astrocytes is ATB^0,+^, which is from the family of neurotransmitters depending on sodium and chlorine ions, coded by the *SLC6A14* gene and transports all amino acids with the exception of aspartate and glutamate [66,67]. ATB^0,+^ may play an important role in the inefficiency of the OCTN2 transporter. It was demonstrated that ATB^0,+^ exists and functions in the apical membrane of brain endothelial cells, creating a blood–brain barrier [68]. Recently, the neutral amino acid transporter coded by the *SLC7A5* gene was reported to also be able to transport L-carnitine, connected with the onset of ASD [69].

Disturbance of cell homeostasis in brain tissue leads to multidirectional disturbances of biochemical parameters, mostly in the cholinergic system. Acetylcholine plays a key role in cognitive brain functions and participates in the regulation of cognitive processes, attention, and memory. The incorrect expression of cholinergic receptors, mainly nicotinic, was confirmed in people with autism [70]. The chemical structures of L-carnitine and its acylcarnitines are comparable to choline and acetylcholine (Ach). Acetyl-L-carnitine positively influences the nervous system because its acyl groups are donated in acetylcholine (Ach) biosynthesis [71]. ALC presents neuroprotective action through the improvement of the energy and function of mitochondria, modulation of gene expressions, antioxidative action, and stabilization of cell membranes (stimulates biosynthesis of proteins and phospholipids of cell membranes). It also prevents cellular death and neuronal damage. ALC favors binding glycocorticoids and nerve growth factor (NGF) in the hippocampus [72]. Additionally, mitochondrial acylcarnitines supply acyl groups, which are used for the acylation of nuclear histones [73]. Acylcarnitines present very high bioavailability compared to L-carnitine because they have a better ability to cross the blood–brain barrier compared to L-carnitine. Therefore, ALC may be administered in much smaller doses than L-carnitine. ALC is recommended to improve central and peripheral nervous system action, improve memory, learning and memorization, increase energy, and improve physical condition and state of mind in therapies of neurodegenerative brain diseases and peripheral neuropathy [74]. Carnitine deficiency may contribute to a reduction in mitochondrial copy number and may disturb neurodevelopment, perhaps specifically affecting neurogenesis or synaptic development [75].

## 5. Role of Carnitine in the Oxidation of Fatty Acids

**Figure 2:**
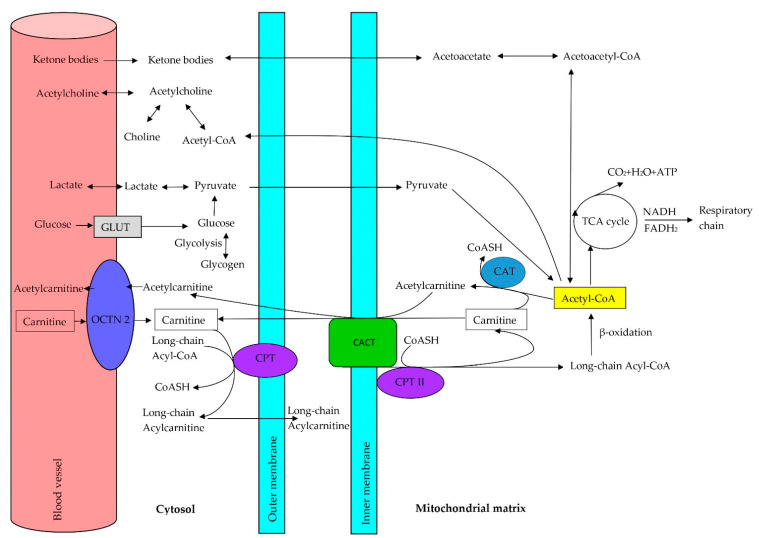
Metabolic roles of carnitine and other substances in the brain. Abbreviations: GLUT, glucose transporters; OCTN2, sodium-dependent carnitine organic cation transporter; CPT I, carnitine palmitoyltransferase I; CPT II, carnitine palmitoyltransferase II; CACT, carnitine-acylcarnitine translocase; CAT, carnitine acetyltransferase; CoASH, coenzyme A; Acetyl-CoA, acetyl coenzyme A; TCA cycle, tricarboxylic acid cycle.

L-carnitine is important in fatty acid metabolism in the brain, although fatty acid oxidation in nervous tissue is less important than glucose oxidation (in normal conditions, brain tissue does not use fatty acids as energy substrates because glucose is the basic brain fuel) [65]. As mentioned earlier, L-carnitine participates in the transport of long-chain acyl groups from the cytoplasm to the mitochondria, thus regulating the concentration of acyl-CoA and CoA in the cytosol and mitochondria and protecting correct cell metabolism [76,77]. In the mitochondria, activated fatty acids are cut into two carbon fragments (acetyl-coenzyme A (acetyl-CoA)), which are oxidized in the tricarboxylic acids cycle (Krebs cycle). Acid groups not removed from the cellular pool inhibit the oxidation of short-chain fatty acids, which results in the peroxidation of lipids in cellular membranes. The deficiency of free CoA limits the efficiency of the Krebs cycle in activating anaerobic glucose metabolism (which inhibits pyruvate dehydrogenase activity, which is responsible for the conversion of pyruvate to acetyl-CoA in aerobic glucose metabolism in cells of the human organism), causing an increase in the concentration of toxic lactate in tissues [58,78,79]. The brain does not oxidize fatty acids directly but uses ketone bodies derived from acetyl-CoA and acetoacetyl-CoA, which are generated by liver β-oxidation of fatty acids. The mitochondrial membrane is not permeable to acyl-CoA, and fatty acids must be connected with L-carnitine to gain entry to the mitochondria interior. L-carnitine creates high energetic ester bonds with long-chain fatty acids catalyzed by carnitine palmitoyltransferase I (CPT-1, CPT I), which is localized in the internal mitochondrial membrane. Three isoforms of CPT-1: CPT-1A, CPT-1B, and CPT-1C [80], were described. CPT-1C is expressed in the liver, brain, kidneys, lungs, spleen, intestines, pancreas, ovaries, and fibroblasts [81]. CPT-1B is a muscular isoform that is strongly expressed in the heart, skeletal muscles, and testicles [81]. CPT1-C is an isoform specific for neurons, but its function in neuronal metabolism remains controversial [82]. Carnitine palmitoyltransferase II (CPT-2, CPT II), localized in the internal mitochondrial membrane, removes carnitine from acylcarnitines and again generates acyl-CoA [81]. After transporting fatty acids from the cytoplasm to the mitochondria, L-carnitine returns to the cytoplasm for the next cycle using carnitine-acylcarnitine translocase (CACT), while acetyl-CoA may enter (under aerobic conditions at low concentrations of ATP) β-oxidation, with the final production of acetyl-CoA (Figure 2) [83].

CPT-1 and CPT-2 are mainly engaged in the import of long-chain acyl-CoA, such as palmitoyl-CoA, oleoyl-CoA, and linoleoyl-CoA, into the mitochondria [81]. Oxidation medium- (C6–C10) and short-chain (C4–C6) fatty acids seem to be independent of the carnitine shuttle [84]. In physiological conditions, long-chain fatty acids are oxidized with the participation of the mitochondrial β-oxidation system, with minimal participation of peroxisomes, where the longest chain fatty acids are oxidized (C ≥ 22). In the peroxisomes, the longest chain FAs are shortened and transported to the mitochondria for β-oxidation and total oxidation to CO_2_ and H_2_O [85]. Fatty acids may be ω-oxidized with the participation of microsomal oxidase, producing dicarboxylic acids. Fatty acids may also be degraded by β-oxidation in peroxisomes to succinate and acetyl-CoA or completely oxidized after transport to the mitochondria in the process of β-oxidation. In physiological conditions, ω-oxidation is a secondary pathway for the oxidation of fatty acids. During disturbances of β-oxidation, the activity of ω-oxidation may increase, generating an excess of dicarboxylic acids. Dicarboxylic acids are unspecific markers of defects in the oxidation of fatty acids [86].

During the incorrect process of fatty acid oxidation, fats released from the fat tissue accumulate in the liver, skeletal muscles, and heart. In the liver, defects in L-carnitine or β-oxidation induce fat deposit and decrease ketone production, which is an alternative source of energy (instead of glucose) for the heart, skeletal muscles, and brain. In the liver, acetyl-CoA allosterically activates pyruvate carboxylase, favoring gluconeogenesis. When fatty acid oxidation is disturbed, fats cannot be used, so glucose is used without regeneration in the cycle of gluconeogenesis because of pyruvate carboxylase inhibition, ketone bodies cannot be produced, glucose level (hypoglycemia) decreases, and brain functioning impairment with loss of consciousness can occur [83].

### Lipid Metabolism Abnormalities Contributing to Autism Spectrum Disorder

Lipids play pivotal roles in the development and function of the human brain and body by acting as regulatory molecules. The literature confirms that altered fatty acid metabolic pathways may be involved in the pathogenesis and expression of autistic traits. The human brain is composed of approximately 60% lipids, with over 20% polyunsaturated fatty acids (PUFAs). PUFAs, predominantly arachidonic acid (AA), eicosapentaenoic acid (EPA), and docosahexaenoic acid (DHA), are the main component of the neural cell membrane phospholipids. DHA and AA play important roles such as in neurogenesis and neuronal differentiation and plasticity, signal transduction, and learning and memory [87]. Considerably, lower levels of PUFAs (DHA, AA, linolenic (ALA), and linoleic acid (LA)) and low L-carnitine concentration are demonstrated in children with ASD in comparison to healthy children. Additionally, ω6/ω3 (AA/DHA) fatty acids serum ratio is significantly higher in children with ASD than in healthy children. A significantly positive correlation (*p* < 0.001) between lower serum L-carnitine concentration and increased ω6/ω3 ratio in the plasma of autistic children was reported [88]. Changes in the composition of polyunsaturated fatty acids in plasma and cell membranes of erythrocytes, especially low concentrations of AA and DHA, were reported in neurological diseases: schizophrenia, attention deficit hyperactivity disorder (ADHD), depression, bipolar affective disease, and ASD. Lowered levels of AA, docosatetraenoic (DTA), and *DHA* in erythrocyte cell membranes in autistic people may be caused by the increased activity of phospholipase type IV in erythrocytes, which suggests possible changes in the phospholipid metabolism of autistic people [89,90,91,92]. Other authors [93] did not observe any deficiency of essential unsaturated fatty acids ω-3 and ω-6 in the erythrocyte cell membranes of ASD children. However, they observed increases in concentrations of eicosanoic acid (20:1n9), erucic acid (22:1n9), and 11,14-eicosadienoic acid (20:2n6), but trans-unsaturated fatty acids 16 and 18 were significantly decreased in regressive autism subjects compared with early-onset autism subjects. The above research was performed in small groups of ASD patients (20 with early autism and 20 with progressive regression), so it could not provide strong evidence for the hypothesis that the incorrect metabolism of fatty acids plays a significant role in the pathogenesis of autism spectrum disorder even though abnormalities in the concentrations of other fatty acids suggested the presence of other metabolic abnormalities in regressive autism [93]. In another study [13], in all children with ASD, increases in the level of polyunsaturated long-chain fatty acids and/or saturated very-long-chain fatty acids (VLCFAs) were observed in comparison to the control group, which suggested disturbed mitochondrial β-oxidation. Increased concentrations of saturated and polyunsaturated VLCFA-containing phosphatidylethanolamines (PtdEtns), DHA-containing ethanolamine plasmalogens (PlsEtns), and DHA-PtdEtns in plasma may cause the excessive microglial activation in the central nervous system (CNS) that is observed in autism. It is thought that impaired mitochondrial fatty acid oxidation is the cause of the elevated plasma levels of VLCFA-containing PtdEtns [13]. Glial cells are vital in cell–cell interactions during nervous cell migration and synaptic plasticity, being necessary for maintaining a stable nervous microenvironment, particularly in periods of increased metabolic stress. Abnormalities in glial functioning may be manifested as a general increase in the thickness of white matter observed in autistic patients [94,95]. A hypothesis was presented that L-carnitine deficiency in the brain may disturb neurogenesis and synapses development. In autism, fatty acid deficiency, covering PUFA and DHA, may be important because brain phospholipids, PUFA, and particularly DHA contribute to the preservation of correct membrane structure and function. Brain phospholipids (PUFA and particularly DHA) participate in eicosanoid signalization, modulation of gene expression and may be markers of disorders in mitochondrial fatty acid oxidation and may favor autism appearance. Therefore, appropriate neonate nutrition may prevent autism appearance [96].

Forty-eight important metabolites have been identified in the plasma of children with ASD as being involved in lipid biosynthesis and metabolism, oxidative stress, and synaptic function. Among these, FAs, such as linolenic acid, EPA, linoleic acid, palmitic acid, oleic acid, myristic acid, acyl-CoA (ACs), 2-aminobutyric acid, and 2-hydroxybutyric acid, were listed as significant metabolites. Among them, increased levels of O-acetylcarnitine and fatty acids (FAs), such as omega-3 and omega-6, were found, which showed correlations with the clinical diagnosis of ASD. A specific reduction in very-low-density lipoprotein (VLDL) concentration, which was correlated with apoprotein A (APOA), and a reduction in apoprotein B (APOB) in the serum of children with ASD were also found [97].

Considering the key role of fatty acids in the correct functioning of cellular membranes and homeostasis in brain cells, a defect in the metabolism of fatty acids (e.g., because of disturbed L-carnitine metabolism) may have important biological implications in autism. Therefore, autistic patients should be additionally diagnosed concerning PUFA deficiency and disorders in PUFA equilibrium; in case of abnormalities, they should be treated with supplements rich in PUFA (vegetable and fish oils). Certainly, further investigations are necessary to better understand the importance of abnormalities in the metabolism of fatty acids in ASD.

## 6. Primary and Secondary L-Carnitine Deficiency

**Figure 3:**
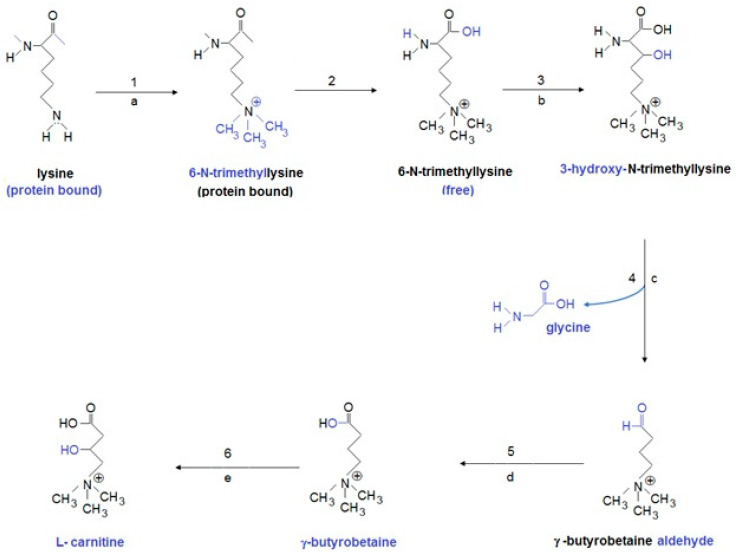
Carnitine biosynthetic pathway. Abbreviation: enzymes: 1, S-adenosyl methionine: ε-N-lysine methyltransferase; 2, protease (lysosomes); 3, ε-N-trimethyllysine dioxygenase (TMLD*) (mitochondria); 4, β-hydroxy-ε-N-trimethyllysine aldolase (HTMLA) (cytosol); 5, 4-N-trimethylaminobutyraldehyde dehydrogenase (TMABA-DH) (cytosol); 6, γ-butyrobetaine dioxygenase (BBD*) (cytosol); second substrates and products: (a) S-adenosyl methionine (SAM)→ S-adenosyl homocysteine (SAH); (b) 2-ketoglutarate + 02 →succinate + C02; (d) NAD+ → NADH + H+; (e) 2-ketoglutarate + 02 → succinate + C02; coenzymes: (b) vitamin C, iron; (c) pyridoxal phosphate; (e) vitamin C, iron. * genetically determined enzyme deficiency.

Disturbed L-carnitine metabolism has recently been connected with neurodevelopment disturbances, including autism spectrum disorder. The L-carnitine biosynthetic pathway includes four enzymes: ε-N-trimethyllysine hydroxylase (TMLD), β-hydroxy-ε-N-trimethyllysine aldolase (HTMLA), 4-N-trimethylaminobutyraldehyde dehydrogenase (TMABA-DH), and γ-butyrebetaine dioxygenase (BBD) (Figure 3).

The OCTN2 transporter (organic cation/carnitine transporter) transports carnitine to the cells. L-carnitine allows the transport of long-chain fatty acids from the cytosol to the mitochondrial matrix by the mitochondrial carnitine–acylcarnitine cycle, which is composed of three enzymes: carnitine palmitoyltransferase I (CPT I), carnitine-acylcarnitine translocase (CACT), and carnitine palmitoyltransferase II (CPT II) (Figure 2). Therefore, autism may be induced by inborn errors in L-carnitine biosynthesis, the transport of L-carnitine to the mitochondria, or the mitochondrial carnitine–acylcarnitine cycle [98]. L-carnitine deficiency creates defects in fatty acid oxidation, which is used to produce energy. Three types of L-carnitine deficiency have been distinguished: primary systemic carnitine deficiency, primary myopathic L-carnitine deficiency, and secondary L-carnitine deficiency. Genetic defects in the enzymes participating in L-carnitine synthesis, defects in the proteins responsible for carnitine transport into cells, or defects in the mitochondrial carnitine–acylcarnitine cycle lead to disturbances in fatty acids metabolism. Recognition of primary carnitine deficiency may be confirmed biochemically by low levels of free carnitine in plasma (<8 μM; reference values: 25–50 μM), caused by lowered renal feedback absorption (<90%) and correct renal function, without abnormality in urinary excretion of organic acids. However, a diagnosis should be confirmed by molecular tests [99].

Clinical symptoms of disorders of biosynthesis or carnitine excretion differ but predominantly cover hypoketotic hypoglycemia, myopathy/cardiomyopathy, and liver insufficiency. Symptoms of disturbances in biosynthesis and excretion of carnitine occur because of energy shortages and the accumulation of fatty acids in some organs. Secondary carnitine deficiency may also occur due to other reasons, e.g., malnutrition, poor absorption, valproic acid toxicity, pivalinic acid present in antibiotics, hemodialysis, and increased loss in urine. Carnitine deficiency connected with low plasma carnitine concentration is less harmful than primary carnitine deficiency and may be treated with low doses of carnitine.

### 6.1. Systemic Primary L-Carnitine Deficiency

Systemic primary carnitine deficiency (SPCD) is a progressive autosomal disturbance connected with impaired carnitine uptake by plasmatic membranes because of a deficiency in the OCTN2 transporter, which is coded by the *SLC22A5* gene (localized on the 5q31 chromosome). Heterozygous or homozygous deficiency of OCTN2 transporters may be an autism risk factor [100,101]. In early life, SPCD is usually recognized as a metabolic decompensation manifested by hypoketotic hypoglycemia; encephalopathy, frequently connected with liver enlargement; increased serum level of aminotransferases; hyperammonemia; cardiomyopathy; muscular weakness; changed intestinal peristalsis and repeated infections in early life. The symptoms of carnitine deficiency may resemble Rey syndrome and may end with the sudden death of the neonate. The large accumulation of lipids in the skeletal muscles, heart, and liver and lowered carnitine concentration in the above-mentioned organs are found in SPCD. Plasma levels of FC, TC, and AC are very low, so autistic children require carnitine supplementation [102,103]. The low concentrations of plasma carnitines in patients with SPCD are caused by excessive urinary carnitine excretion because of defective reversible absorption in renal tubules; therefore, the carnitine level in plasma and tissues (heart and skeletal muscles) may drop to below 10% of normal values. In the muscles of patients with SPCD, the total carnitine content may be very low, even less than 5% in comparison to healthy people [102,103,104]. Basic treatment of SPCD patients involves carnitine supplementation, which should be introduced as soon as possible after diagnosis before the appearance of irreversible damage to the internal organs [100]. Monocarboxylic acid transporter 9, coded by the *SLC16A9* gene, which transports carnitine from renal tubules to plasma, may be important. The deficit in this gene was not reported in SPCD patients, but it was reported in numerous individual polymorphisms, including *RS7094971*, which is associated with abnormal plasma levels of FC and AC [105].

Detection of frequent inborn errors in the biosynthesis of carnitine from trimethyllysine (Figure 2), caused by a deficiency in the activity of the *TMLHE* gene, may help explain the reasons for dysmorphic autism. *TMLHE* (conjugated with chromosome X), which codes trimethyllysine dioxygenase (TMLD), the first enzyme on the mitochondrial carnitine synthesis pathway, was detected in men with nondysmorphic autism (frequency 1 in 350 men). However, only about 3% of men with *TMLHE* gene deficiency developed autism. The risk of nondysmorphic autism connected with *TMLHE* mutation may be diminished by appropriate carnitine supplementation during the early stages of child brain development [11,12,96,106]. Butyrobetaine dioxygenase (BBD) is the next false enzyme participating in carnitine biosynthesis, coded by the *BBOX1* gene, which may induce autism [98]. Symptoms of carnitine deficiency (small head, delay in speech, tenuous growth, and presence of some dysmorphic traits) in a girl with a homozygous deletion of *BBOX1* were described by Rashidi-Nezhad et al. [107]. Clinical symptoms of carnitine deficiency were not detected in the presented girl, but laboratory tests showed plasma FC concentration was at the lower limit of the reference values. Acylcarnitine profile was also within the reference ranges, and the free carnitine/acylcarnitine ratio was in the normal range in this girl. The authors suggested that dietary carnitine consumption and renal reabsorption were sufficient for correct carnitine homeostasis in the above-described case [107]. Recently, Lee et al. [108] showed that expression of *BBOX1* is decreased in the mouse model of schizophrenia. They tested 284 people in the Korean population with schizophrenia and 409 healthy people and found that *BBOX1* polymorphisms may be connected with increased schizophrenia susceptibility in this population. As mentioned earlier, the mitochondrial carnitine–acylcarnitine cycle is composed of three enzymes: CPT-1, particularly neuronal isoform CPT-1C (localized in the hypothalamus, almond body, and hippocampus, which plays an important role in neurological and neuropsychiatric disorders); CACT; and CPT-2., We did not find data on defects concerning the above-mentioned enzymes in autistic children in the literature.

### 6.2. Secondary L-Carnitine Insufficiency

The clinical consequences of secondary L-carnitine deficiency, even though it occurs more frequently than primary L-carnitine deficiency, are less harmful than those of primary L-carnitine deficiency. A diet poor in L-carnitine (e.g., vegetarian diet), malnutrition, absorption and transport disturbances, increased urinary carnitine excretion, liver diseases (cirrhosis), chronic renal diseases, and administration of some drugs (e.g., antiepileptic drugs, including valproic acid and carbamazepine, phenytoin, phenobarbital, beta-lactam antibiotics, and anticancer drugs) may be reasons for secondary L-carnitine deficiency. The above-listed drugs are excreted in the urine in combination with L-carnitine, thus lowering the carnitine plasma concentration. The above-mentioned drugs may also inhibit OCTN2 transporters, leading to secondary carnitine deficiency [109,110]. Secondary carnitine deficiency may result from defects in any enzyme participating in the mitochondrial oxidation of fatty acids because excess fatty acids linked to carnitine are excreted into the urine as acylcarnitines. Therefore, with secondary carnitine deficiency, the free carnitine pool is shifted in the direction of acylcarnitines because carnitine is used to eliminate the acyl groups of the accumulated fatty acids resulting from the dysfunction of fatty acids mitochondrial metabolism. Normal or slightly lowered FC levels are present in plasma, but AC levels and AC/FC ratio are increased [111,112]. Enzymatic defects in fatty acid oxidation may concern very-long-chain fatty acids acyl-CoA dehydrogenase (VLCAD), medium-chain acyl-CoA dehydrogenase (MCAD), long-chain 3-hydroxyacyl-coenzyme A dehydrogenase deficiency (LCHAD), CPT-2, and CACT deficiency, which may cause secondary carnitine deficiency [109,113]. It was demonstrated that defects in the activity of long-chain acyl-CoA dehydrogenase (LCAD) when oxygenizing long-chain fatty acids or dehydrogenase deficiency may cause autism. Significant increases in the concentration of unsaturated fatty acids C14:1 and C14:2 were reported in ASD patients. The acylcarnitine profile change of ASD patients is similar to the change observed in experimental mice with a deficit in LCAD activity [114]. Disturbances in the metabolism of amino acids, e.g., isovaleric acidemia, methylmalonic acidemia, or glutaric aciduria type I, may be another reason for secondary carnitine deficiency [115]. In primary carnitine deficiency, the plasma free carnitine concentration is below 5 μM/L, but plasma free carnitine concentration in secondary carnitine deficiency is higher and may amount to a little below 20 μM/L, which is accompanied by a normal tissue concentration of carnitine. Supplementation with carnitine in secondary carnitine deficiency requires small carnitine doses because carnitine levels may be normalized quickly. As carnitine biosynthesis provides only 25% of the carnitine pool, defects in carnitine biosynthesis are not the reason for carnitine deficiency in people with normal renal function and a regularly balanced diet. However, limited consumption of carnitine may cause its deficit, especially in neonates (in parenteral nutrition or during the administration of milk substitutes without carnitine). Additionally, in neonates, endogenic carnitine synthesis is limited because of the immaturity of liver enzymes, particularly γ-butyrobetaine dioxygenase (BBD), and the immaturity of renal canaliculus, which decreases the kidney’s carnitine reabsorption ability [7]. Laboratory tests in ASD patients demonstrated a disturbed acylcarnitine profile, which may be potential biomarkers reflecting the existence of acquired mitochondrial disease. The secondary FC deficiency is connected with the increased concentration of AC and AC/FC ratio [111].

## 7. L-carnitine A Potential Biomarker of Mitochondrial Disturbances

Free carnitine transports long-chain fatty acids from the cytoplasm to the mitochondria as acylcarnitines. Acylcarnitines undergo β-oxidation, producing energy in the mitochondria. There are short-(3–5), medium- (6–12), and long-chain (>12 carbon atoms) fatty acids. Defects in the L-carnitine shuttle (L-carnitine is released from fatty acid and creates acetyl-CoA) in the mitochondria mainly disturb the oxidation of long-chain fatty acids, as a medium- and short-chain fatty acids may directly penetrate mitochondrial membranes without L-carnitine mediation. Abnormal fatty acid oxidation has been observed in ASD: (1) A low-level of free carnitine (main cofactor in the transport of long-and very-long-chain fatty acids from the cytoplasm to the mitochondrial matrix) was observed in ASD children because of lowered mitochondrial β-oxidation of fatty acids. (2) Increases in the concentrations of long- and very-long-chain fatty acids were detected in the serum of ASD children in comparison to the control group, which suggested an excess of unprocessed fatty acids. (3) In ASD patients, a significant increase in the concentration of acylcarnitines was detected. These data suggest the existence of abnormalities in fatty acids metabolism with the participation of L-carnitine [14]. It is possible that mitochondrial defects are caused by L-carnitine deficiency and blockade of secondary fatty acid oxidation, as low serum concentrations of free and total carnitine were observed in children with ASD in comparison to the control group (*p* < 0.001) [116]. Children with ASD had considerably lowered concentrations of short- and long-chain acylcarnitines compared with medium-chain acylcarnitines, which suggested the presence of disturbances in L-carnitine circulation and mitochondrial dysfunction [117]. The observed carnitine deficiency in ASD patients, accompanying lactate increase in the blood, and the significant increases in alanine and ammonia levels point to mild mitochondrial dysfunction [111,116]. The results published by Rossignol and Frye indicated the presence of low total carnitine plasma concentrations in up to 90% of the investigated ASD children [38].

Accurate and precise determination of free carnitine and individual acylcarnitines became possible after the introduction of new diagnostic techniques such as mass spectrometry. Characteristic acylcarnitines profiles observed in disturbances connected with mitochondrial defects in people with autism spectrum disorder may be promising biomarkers of primary and secondary carnitine deficiencies. Abnormalities in the acylcarnitine profiles in ASD patients, such as abnormally increased acylcarnitine panel C4OH, C14, C16:1, and C16 in 74 (35%) of 213 investigated patients, confirmed the results of Frye et al. [14]. Incorrectly increased acylcarnitines concentrations (hydroxybutyryl carnitine, myristoyl carnitine, and enoylpalmitoyl carnitine) were found in 17% of the investigated children with ASD [14]. Lv et al. [117] reported low concentrations of free carnitine and acylcarnitines (glutarylcarnitine, octylcarnitine, and carnosylcarnitine) in children with ASD in dry blood spots in comparison to healthy children. The diagnostic accuracy, analyzed using the receiver operating characteristic (ROC), showed 93% specificity and 40% sensitivity (area under the curve (AUC) = 0.72) for glutarylcarnitine. Diagnostic accuracy analyzed with ROC showed 80% specificity and 50% sensitivity (AUC = 0.66) for free carnitine and 80% specificity and 50% sensitivity (AUC = 0.66) for carnosylcarnitine. The profiles of carnitine and acyl-carnitines change significantly during the first year of life but remain at the same level between 2 and 15 years of age. Therefore, determining the profiles in a dried blood spot can provide an early indication of higher classification efficiency (sensitivity 72.3%, specificity 72.1%) [48]. The lowered amounts of free carnitine and short- and long-chain acylcarnitines in children with ASD suggest potential mitochondrial dysfunction and the pathologic metabolism of fatty acids. Serum concentrations of glutarylcarnitine and carnosylcarnitine may be potential biomarkers in ASD diagnostics, as suggested by Lv et al. [117]. Disturbances in carnitine metabolism may be important in the diagnosis of nondysmorphic autism, as suggested by Ratajczak and Sothern [118]. The experimental research conducted by MacFabe [119] in rats demonstrated changes in the composition of phospholipids during the induction of autistic behavior after intra-intestinal infusion of propionic and butyric acids, which is similar to ASD in people. It appeared that intra-intestinal infusions of propionic acid increased the motor activity of experimental rats. Additionally, propionic acid increased the immune reactivity of monocarboxylate transporter 1 (mainly in the external bag of white matter), which suggested changes in both transport and metabolism of short-chain fatty acids in the brain. Propionic acid and butyric acid (to a lesser degree) in all phospholipid classes lowered the level of all monounsaturated fatty acids, all ω-6 fatty acids, and saturated fatty acids. The general amount of acylcarnitines, long-chain acylcarnitines (C12:24), short-chain acylcarnitines (C2:9), and the ratio of bound to free carnitine increased after experimental infusions of propionic and butyric acids. Evidence indicating the relationship of ASD with processes in the alimentary tract is increasing due to intestinal microflora, intestinal bacterium cell membrane components (lipopolysaccharides), or intestinal bacteria metabolites. Propionic acid produced by Clostridia, Bacteroides, and Desulfovibri, during carbohydrates fermentation or presence in the diet may disturb the functioning of the GABA-ergic system, which plays a significant role in autism pathogenesis [5,95,119]. Lowered concentrations of free and total carnitines in the plasma of autistic children are connected with gastrointestinal symptoms. L-carnitine deficiency, accompanied as mentioned earlier by a slight increase in lactate and significant increases in alanine and ammonia, is a symptom of mitochondrial dysfunction. Determination of blood acylcarnitine profiles may be a biomarker of mitochondrial disturbances in fatty acid oxidation.

## 8. Dietary Supplements May Reduce Autism Symptoms

Elaboration of new therapies improving both mitochondrial function and clinical symptoms of ASD is extremely important. Identification of pathophysiological anomalies connected with ASD in individual patients may lead to a better understanding of the dysfunctions related to an individual patient and may lead to the provision of individual treatment. Autism treatment should start as soon as possible, including psychotherapy, pharmacotherapy, and appropriate diet and supplementation. Administration of diet supplements (amino acids, fatty acids, vitamins/minerals, and probiotics) in autistic children seems to be a safe procedure for supporting therapy. Literature data concerning diet supplements in ASD are controversial, and earlier research on diet supplementation in ASD presents significant methodological heterogeneity [120]. Except for innovative mitochondrial therapies, presently used strategies include the addition of antioxidants to the diet such as L-carnitine, coenzyme Q10, and mitoquinone mesylate (MitoQ_10_); other mitochondria-targeted antioxidants including N-acetylcysteine (NAC); vitamins C, E, K1, and B; and sodium pyruvate or lipoic acid [6,121,122]. Of physicians, 49% recommend vitamin/mineral supplements for ASD patients because numerous reports show that vitamin/mineral supplementation is beneficial to improving nutrition status and metabolism as well as reducing autistic symptoms. Therefore, vitamin/mineral supplementation may be considered supportive therapy for the majority of autistic children and adults. Appropriate adjustment of the diet to ASD progress may have positive effects on silencing and attenuation of the psychical and gastroenterological symptoms occurring in autism [123,124].

### 8.1. L-Carnitine and Acetyl-L-Carnitine Supplementation in ASD

Supplementation of carnitine to alleviate behavioral and cognitive symptoms in ASD patients due to the deficit is absolutely necessary as a potential treatment method. In the case of primary carnitine deficiency, ASD treatment with L-carnitine/acylcarnitine earned the high assessment of B (in the ranking from A, solid proofs, to D, limited, incoherent, or ambiguous proofs) [6,34]. The first randomized clinical trial concerning results of L-carnitine treatment in patients with diagnosed autism spectrum disorder was performed in 2011 [125]. A total of 27 autistic children were divided into two groups: one group (16 people with ASD) received L-carnitine at a dose of 50 mg/kg body mass/day for 3 months, and the other group (11 people) receiving a placebo and were tested in the above trial. After three months, the children receiving L-carnitine presented reductions in autism symptoms. Furthermore, increases in muscular force and improvements in cognitive functions were connected with the higher level of free blood L-carnitine [125].

The influence of higher L-carnitine dose (100 mg/kg body mass/day) over 6 months in 35 patients with ASD was evaluated in the next randomized clinical trial, and a significant improvement in ASD symptoms was observed. Concentrations of free and total L-carnitine in dry blood drop increased more significantly after six months than after three months of L-carnitine treatment; a positive correlation of the applied carnitine doses with cognitive and behavioral status in autistic children during six months of L-carnitine therapy was demonstrated [106]. An 8 week experiment of oral supplementation with a suspension or pills of carnitine in three divided doses starting from 200 mg/kg increasing to 400 mg/kg day, to a maximum of 6 g daily in 10 boys with ASD (including one patient with genetic ASD with a deficiency in *TMLHE* gene) was described in Goin-Kochel et al. [126]. They presented proof that high doses of L-carnitine, up to 400 mg/day, are safe, although mild diarrhea and atypical body smell (fishy smell) were present as the only adverse effects. Careful and detailed psychological observation of the boys for four and eight weeks revealed the efficacy of L-carnitine treatment because significantly positive correlations between serum increased free and total L-carnitine levels and improvement in cognitive functions were found.

Acetyl-L-carnitine applied for 12 months at a dose of 50 mg/kg body mass/day (maximum to 2 g acetyl-L-carnitine/day) was beneficial and well-tolerated. Adams et al. [127] found a significant increase in plasma L-carnitine concentration in the treated people in comparison to the untreated group. However, the increase in acylcarnitine plasma concentration was not significant in the treated group in comparison to the non-treated group, suggesting mutual conversion between both carnitine forms [127]. Adams et al. suggested that the absorption of acetyl-L-carnitine was worse than that of free L-carnitine, so it may be less effective. The above-described research found an increase in the total carnitine plasma concentration, but only by 25%, which less than reported by Geier et al. [125], where an equivalent L-carnitine dose was applied and a 70% increase in the total plasma carnitine was observed. They also observed that L-carnitine application at a dose of 50 mg/kg body mass/day for three months significantly improved the cognitive function of ASD patients. Torioli et al. [128] stated that acetyl-L-carnitine at a dose of 20–50 mg/kg/day (for 12 months) decreased irritability and improved the social behavior of young boys with breakable X chromosomes, where ASD symptoms occurred in more than 60% cases. Torioli et al. recommended acetyl-L-carnitine as an effective treatment for ADHD in children with breakable X chromosomes, particularly in the group of children that do not tolerate stimulating drugs [128].

A positive influence of L-carnitine treatment in people with genetically determined ASD was described in the review of Malaguarnera and Cauli [129]. Evidence was presented that L-carnitine administration was beneficial in the treatment of metabolic irregularities in some genetic disturbances that are commonly connected with ASD. A 4-year-old child with *TMLHE* gene mutation, which codes ε-N-trimethyllysine dioxygenase (TMLD) enzyme, which participates in carnitine biosynthesis, was treated with L-carnitine at 200 mg/kg/day for 4.5 months and demonstrated observable gradual declines in ASD symptoms. The child’s parents noticed a significant improvement three months after starting the carnitine supplementation, including directly looking at the parents and smiling, better visual contact, increased attention, and awareness and interest in other people. An increase in plasma free acylcarnitines was found three months after starting carnitine supplementation [12]. A significant improvement in health was reported in three patients with mitochondrial disease connected with respiratory chain II–IV who were treated with 50 mg/kg/day with L-carnitine and complex vitamins of the B group, coenzyme Q10, and folic acid [130]. It was proven that patients with early carnitine treatment and without frequent metabolic decompensations were less vulnerable to the development of pathological, neurological, and neuropsychological attributes, including ASD, in research concerning eight propionic acidosis patients with autistic symptoms (e.g., repeated movements or hand fluttering, stereotypic behavior, disturbances in social interactions, difficulty in communication with other people), who were treated with L-at 50 mg/kg/daily, biotin at 10 mg/day, and a low-protein diet [131]. Significant ketogenesis, diminished by L-carnitine supplementation, clearly lowered oxidative stress parameters (e.g., reduction in malonic dialdehyde concentration) in patients with propionic or methylmalonic acidosis during starvation [132,133]. Reports have indicated a clinical improvement in patients with other acidoses, e.g., glutaric acidemia type I (deficiency in glutaryl-CoA dehydrogenase), after L-carnitine treatment. Glutaric acidemia type I is characterized by the accumulation of glutaric acid (GA), 3-hydroxyglutaric acid (3-OH-GA), glutaconic acid, and glutarylcarnitine (C5DC) in serum and urine. Patients with glutaric acidemia type I should be supplemented with L-carnitine for their whole life to maintain correct concentrations of plasma L-carnitine [134,135]. Further reports proved that L-carnitine supplementation as a complementary therapy contributes to improvements in many blood markers of oxidative damages (activities of catalase, superoxide dismutase, and glutathione peroxidase) in phenylketonuria (PKU) patients. The results of the above-presented research suggested that L-carnitine and selenium supplementation may partially mitigate neurological symptoms occurring in PKU patients [136]. L-carnitine supplementation may act as a therapy in other diseases, e.g., in maple syrup disease (MSUD), where patients have carnitine deficiency [137,138], in systemic primary carnitine deficiency (one girl with mutation of *SCL22A5* gene treated with L-carnitine at 200 mg/kg/day), and in vitamin B complex, resulting in improvements in muscular strength as well as language abilities: IQ was improved by 15%. A slight improvement in autistic attributes was also observed [100]. Supplementation with L-carnitine, coenzyme Q10, omega-3 fatty acids, and multivitamins in 4q-syndrome, which is characterized by slight face and cranial dysmorphism, finger abnormalities, defects in heart and bone systems, as well as mental retardation, where 33% of these patients had attributes of ASD, showed improvement in mood, increased energy and muscular strength, as well as better speech development and expressing oneself [139]. Benefits of L-carnitine and other supplements (vitamins of B group, coenzyme Q10) application include improvements in ASD symptoms, augmentation of the immune system, and increased levels of energy and muscular strength were observed in the above-listed genetic disturbances connected with ASD [69,129]. Therefore, it is reasonable to evaluate L-carnitine consumption in the diet and consider the use of L-carnitine supplementation because high fats consumption in the diet means that more fatty acids must be transported to the mitochondria for oxidation. The transport of long-chain fatty acids from the cytoplasm to the mitochondria demands the presence of L-carnitine, which increases the risk of exhaustion of carnitine resources in an organism, especially in ASD patients with low L-carnitine levels. L-carnitine supplementation is necessary during the treatment of ASD children with valproic acid because L-carnitine supplementation decreases hyperammonemia in patients with encephalopathy induced by valproic acid. L-carnitine supplementation is particularly recommended in cases of heavy valproate intoxication. This is why serum L-carnitine concentration should be monitored during valproic acid application to ASD children. Therefore, L-carnitine supplementation, particularly in situations where patients consume small amounts of beef or pork, should be started in people with ASD. Oral doses of L-carnitine can range from 30 to 100 mg/kg/day in two or three divided doses, which is recommended for ASD people [34].

### 8.2. L-Carnitine Supplementation during Pregnancy

The lowered availability of L-carnitine during pregnancy is a frequent cause of ASD appearance in neonates. Therefore, women planning a pregnancy or who are pregnant should eat a suitably balanced diet enriched in vitamins, minerals, and L-carnitine [8]. The recommended amount of L-carnitine for pregnant women is 1500–2000 mg daily in 3–4 doses, taken with food rich in L-carnitine and iron [140]. L-carnitine application in adults for improvement in brain function, lowering blood pressure, and improvement in heart function indicates that even daily consumption of 2 g of L-carnitine does not create undesirable effects. According to Keller et al. [141], daily doses of 500 mg L-carnitine are sufficient to prevent deficiency of L-carnitine during pregnancy without side effects for the mother and fetus [141]. L-carnitine occurring in natural food, mostly in red meat, whereas other meats and dairy products contain less L-carnitine, should be considered when planning L-carnitine supplementation [142,143,144,145] (Table 1). Notably, absorption of L-carnitine in foods containing L-carnitine is much higher (54% to 87%) than from dietary supplements, where absorption is only about 15% [69].

## 9. Conclusions

Autistic disturbances may be connected with impairment of mitochondrial functions. Biomarkers of mitochondrial dysfunction associated with ASD are direct markers, e.g., lactate, pyruvate, ubiquinone, alanine, and acylcarnitine profiles, and indirect markers such as creatine kinase ALT, AST, ammonia, free L-carnitine and total L-carnitine. Free L-carnitine and acylcarnitine profiles in dried blood spots may be potential biomarkers of the pathological mitochondrial metabolism of fatty acids in patients with ASD. The relationship between carnitine and autism has been well-documented. Primary defects in carnitine synthesis or transport (deficiency OCTN2, trimethyllysine dioxygenase, butyrobetaine dioxygenase) are observed in about 10–20% of ASD patients. Plasma levels of free L-carnitine, total L-carnitine, and acylcarnitines are very low in children with systemic primary carnitine deficiency (SPCD). Secondary L-carnitine deficiency, having different etiologies (lowered L-carnitine content in the diet, disturbances in absorption and transport, increased urinary excretion, and diseases of liver or kidneys, occurs more frequently but is less harmful than primary carnitine deficiency. Characteristic abnormalities of free L-carnitine, total L-carnitine, and acylcarnitines profiles observed in disturbances connected with mitochondrial defects in people with autism spectrum disorder may be promising indirect biomarkers of primary and secondary carnitine deficiencies.

Research on diet and supplementary L-carnitine administration demonstrated improvement in neurologic and neuropsychiatric functions of ASD patients. L-carnitine therapy not only significantly increases serum L-carnitine concentration but also has positive correlations between the increase in serum L-carnitine and the reduction of specific autistic symptoms. Additionally, the combination of L-carnitine treatment with adequately composed vitamins, minerals, and essential fatty acids in ASD may help improve mitochondrial functions and may provide substantial clinical benefits. Mitochondria may be restored and regenerated, and L-carnitine supplementation may support the functioning of mitochondria.

Due to the comprehensive understanding of the biological basis of autism disturbances, in the future, it will be possible to prepare detailed personalized and precise programs of pharmacological ASD treatment with the help of new drug therapies, with appropriately chosen diet supplements to obtain optimal therapy results. Further research is required to establish the role of carnitine metabolism in neurodevelopmental disorders like ASD, as well as the efficiency of carnitine supplementation in patients with these disorders.

## Figures and Tables

**Table 1:** The amount of L-carnitine in product groups.

Type of Food	Total L-Carnitine Content
**Ruminant Meat**	**(mg/100 g)**
Kangaroo	637
Horse	423
Ram, tenderloin	162.8
Ram, rump	168.5
Beef steak	232
Beef kidneys	31.0
Beef liver	15.6
Sheep, skeletal muscle	209
Sheep, heart	58.9
Sheep, liver	2.17
Goat	95.0–99.0
Pork	20.0–30.0
Pork liver	10.7
Rabbit, muscle	21.0
Rabbit, liver	11.1
**Poultry**	**(mg/100 g)**
Duck	73.0
Pigeon	52.8
Turkey	51.0
Chicken	34.0
Quail	29.1
Pheasant	13.5
**Fish**	**(mg/100 g)**
Salmon	5.96
Zebrafish	2.80–8.95
Yellow catfish	5.93
**Milk**	**(mg/100 mL)**
Sheep	10.2–12.7
Goat	4.50–7.50
Cow	7.80–9.60
**Milk products**	**(mg/100 g)**
Yogurt	40.0
Buttermilk	38.0
Cottage cheese	22.5–26.6
Sour cream	19.7
Coffee cream	16.6
Cheese	14.0–28.0
**Mushrooms**	**(mg/100 g)**
Oyster	53.0
Champignon	29.8
Chanterelle	13.3
Other	1.00–6.00
**Vegetables, cereals, and seeds**	**(mg/100 g)**
Cucumber	4.45
Cauliflower	3.26
Carrot	3.73
Maize	0.68
Peas	0.60
Wheat, germ	1.06
Wheat, seeds	0.61–1.22
Peanut	0.10–0.76
**Fruit**	**(mg/100 g)**
Avocado	1.72
Guava	0.82
Banana	0.39
Apple	0.29
Orange	0.22

## Data Availability

Not applicable.
